# Argan Oil as a Rich Source of Linoleic Fatty Acid for Dietetic Structured Lipids Production

**DOI:** 10.3390/life11111114

**Published:** 2021-10-20

**Authors:** Tiago Simões, Jessica Ferreira, Marco F. L. Lemos, Ana Augusto, Rafael Félix, Susana F. J. Silva, Suzana Ferreira-Dias, Carla Tecelão

**Affiliations:** 1 MARE-Marine and Environmental Sciences Centre, ESTM, Politécnico de Leiria, 2520-641 Peniche, Portugal; tiago.simoes@ipleiria.pt (T.S.); jessica_pinfer@yahoo.com (J.F.); marco.lemos@ipleiria.pt (M.F.L.L.); ana.l.augusto@ipleiria.pt (A.A.); rafael.felix@ipleiria.pt (R.F.); susana.j.silva@ipleiria.pt (S.F.J.S.); 2 CDRSP-Center for Rapid and Sustainable Product Development, Politécnico de Leiria, 2430-028 Marinha Grande, Portugal; 3 Department of Food and Nutritional Sciences, University of Reading, Whiteknights, Reading RG6 6AP, UK; 4 LEAF, Linking Landscape, Environment, Agriculture and Food, Instituto Superior de Agronomia, Universidade de Lisboa, 1349-017 Lisboa, Portugal; suzanafdias@isa.ulisboa.pt

**Keywords:** *Argania spinosa* oil, capric acid, caprylic acid, commercial immobilized lipases, low-calorie structured lipids

## Abstract

Argan oil is rich in long-chain unsaturated fatty acids (FA), mostly oleic and linoleic, and natural antioxidants. This study addresses the production of low-calorie structured lipids by acidolysis reaction, in a solvent-free system, between caprylic (C8:0; system I) or capric (C10:0; system II) acids and argan oil, used as triacylglycerol (TAG) source. Three commercial immobilized lipases were tested: Novozym^®^ 435, Lipozyme^®^ TL IM, and Lipozyme^®^ RM IM. Higher incorporation degree (ID) was achieved when C10:0 was used as acyl donor, for all the lipases tested. Lipozyme^®^ RM IM yielded the highest ID for both systems (28.9 ± 0.05 mol.% C10:0, and 11.4 ± 2.2 mol.% C8:0), being the only catalyst able to incorporate C8:0 under the reaction conditions for biocatalyst screening (molar ratio 2:1 FA/TAG and 55 °C). The optimal conditions for Lipozyme^®^ RM IM in system II were found by response surface methodology (66 °C; molar ratio FA/TAG of 4:1), enabling to reach an ID of 40.9 mol.% of C10:0. Operational stability of Lipozyme^®^ RM IM in system II was also evaluated under optimal conditions, after eight consecutive 24 h-batches, with biocatalyst rehydration between cycles. The biocatalyst presented a half-life time of 103 h.

## 1. Introduction

Lipids are important diet constituents due to their high energy value and essential fatty acid content, being involved in crucial mechanisms such as cell signalling and hormonal regulation [1]. Triacylglycerols (TAG), the main lipid components of dietary fat, consist of a glycerol backbone linked to three fatty acids. Depending on their structure and composition, they present distinct characteristics such as melting behaviour, digestion, absorption, and metabolic properties [2,3].

Structured lipids (SL), also known as “taylor-made fats”, usually consist of TAG with a chemically or enzymatically modified structure, obtained by changing the original fatty acid (FA) profile and/or their original position in the glycerol structure [4,5,6]. Enzymatic synthesis of structured lipids presents several advantages in comparison with the chemical route namely milder reaction conditions, enantio- and regioselectivity, less toxicity and oxidation, as well as easier product recovery, among others [3,7,8]. Despite not being characteristic for all structured lipids, some of these modified molecules have demonstrated their nutritional and functional properties in treating and preventing particular human diseases [1,9]. In recent years, research focused on low-calorie (dietetic) fats has increased, to meet consumers' demand for a healthier lifestyle and to reduce obesity. Structured triacylglycerols presenting low caloric value comprise TAG containing short or medium-chain FA (C_8_–C_12_), preferably esterified at external *sn*-1,3 positions, and long-chain FA (L, ≥ C_14_) occupying the internal position of the glycerol backbone. These structured lipids, named MLM, provide health and nutrition benefits since they may control various metabolic disorders, such as obesity and fat malabsorption [2,10,11]. The average caloric value reported for these fats is 5 kcal g^–1^, while conventional counterparts present 9 kcal g^–1^ [6,12,13,14]. During digestion, the *sn*-1,3-regioselective pancreatic lipase releases the medium-chain FA from TAG, which are preferentially transported to the liver where they are promptly metabolized via mitochondrial β-oxidation, providing an energy source without accumulation in the adipose tissue [15]. The effective incorporation of the resulting *sn-*2-monoacylglycerols (2-MAG) into chylomicrometers and its absorption through the lymphatic system is observed [7].

MLM structured lipids are currently synthesized by acidolysis reaction between an oil rich in long-chain FA (e.g., olive, olive pomace, linseed, spent coffee grains, grapeseed, avocado, or microbial oils) and medium-chain FA, namely octanoic (C8:0; common name—caprylic acid) or decanoic (C10:0; common name—capric acid) acids, catalyzed by *sn*-1,3-regioselective lipases [10,12,13,16,17,18,19,20,21,22,23,24].

Lipases (E.C. 3.1.1.3., triacylglycerol acylhydrolases) are versatile enzymes able to catalyze the hydrolysis of ester bonds in the aqueous phase and promote the reverse reaction of esterification, as well as interesterification and transesterification reactions, in medium with low water activity [25].

Several immobilized lipases have been tested and used for MLM production, presenting high yields and operational stability in solvent-free systems [4,13]. Implementing cost-effective reaction systems for MLM production may be attained by the use of less expensive biocatalysts, with high activity and operational stability, as well as oils with valuable properties to be used as a TAG source [26,27,28]. In this sense, virgin argan oil may be a feasible substrate due to its anti-inflammatory, antioxidant, and cardiovascular-protective properties. Argan oil (AO) contains natural antioxidants, namely tocopherols and other sterols and phenolic compounds [29,30,31,32]. These compounds provide higher oxidative stability to the argan oil when compared to other oils [33]. They may also be beneficial in the reaction medium, by preventing or delaying lipid oxidation. TAG of argan oil mainly contain oleic (O, C18:1 n-9), linoleic (L, C18:2 n-6) and palmitic (P, C16:0) fatty acids. Therefore, the main TAG are OOL, POL, OLL and OOO [34]. The TAG structure, containing mainly oleic or linoleic acid at position *sn*-2, and the antioxidant composition turn this oil very appealing to be used in structured lipids synthesis, namely, for MLM production.

This study aimed at producing MLM by lipase-catalyzed acidolysis between medium-chain FA (caprylic or capric acids) and TAG from argan oil, in the absence of organic solvents. Three commercial biocatalysts were tested for this purpose. The lipase showing the highest activity was selected for modelling and optimization of reactions conditions (molar ratio (FA/TAG) and temperature).

## 2. Materials and Methods

### 2.1. Biocatalysts, Substrates, and Chemicals

The immobilized thermostable lipases from *Candida antarctica* lipase b (Novozym^®^ 435), *Rhizomucor miehei* (Lipozyme^®^ RM IM) and *Thermomyces lanuginosus* (Lipozyme^®^ TL IM), were kindly provided by Novozymes, A/S, Bagsvaerd, Denmark. Edible virgin argan oil (Emile Noël, France) was acquired from a biological food store. Caprylic (>98% purity; MW = 144.21 g mol^−1^) and capric (>98% purity; MW = 172.26 g mol^−1^) fatty acids, and methyl myristate standard (99%) were acquired from Fluka. Silica-gel 60 (0.25 mm width, 20 × 20 cm) thin layer chromatography (TLC) plates, acetyl chloride, methanol and *n*-heptane were acquired from Merck, Germany, and 2′,7′-dichlorofluorescein was from Sigma-Aldrich. Fatty acid methyl ester mixes (PUFA No 1 from Marine source and PUFA No 3 from Menhaden oil) were acquired from Supelco (Bellefonte, PA, USA).

### 2.2. Biocatalyst Screening

Acidolysis reactions were carried out batchwise, for all the tested biocatalyst, using cylindrical jacketed glass reactors (20 mL) operating at 55 °C, under magnetic stirring of 300 rpm. Rubber caps were used to seal the reactors to minimize oxidation in the reaction system. The reaction medium comprised 1.95 g of AO, as the TAG source, and 1.35 g of C8:0 (system I) or C10:0 (system II) fatty acids, resulting in a molar ratio FA/AO of approximately 2:1, calculated based on the molecular weight of trilinolein. This is the stoichiometric ratio required for the esterification of FA at the external positions of the glycerol backbone by *sn*-1,3 selective lipases. A 5% (*w*/*w*) lipase load was used, with respect to the amount of oil. After 24 h reaction, the enzyme was recovered from the medium by filtration (Whatman™ Grade 1 filter paper) at room temperature. All assays were carried out in duplicate. The filtrate was stored at −20 °C for subsequent analyses.

### 2.3. Time-Course Experiments

The biocatalyst presenting the highest acidolysis activity was used in a time-course reaction to evaluate when a quasi-equilibrium was reached. Thus, an acidolysis reaction between AO and C10:0 (system I), catalysed by Lipozyme RM IM, was performed for 48 h, with withdraw samples along the reaction. The assay was carried out under the same conditions as described in Section 2.2, corrected to a total reaction volume of 7 mL. Duplicate aliquots of 200 µL were collected, under stirring, in every sampling for TAG composition analysis.

### 2.4. Modelling Acidolysis and Optimization of Reaction Conditions

For acidolysis modelling and reaction conditions optimization, as a function of the molar ratio FA/AO and temperature, a set of experiments was performed under the conditions dictated by a central composite rotatable design (CCRD) [35]. In this experimental design, each variable (molar ratio and temperature) was tested at five different levels to determine the effects of each one on MLM production. Experiments were conducted using Lipozyme RM IM lipase for 24 h, following the methodology described in Section 2.2. The lipase load was kept constant at 5% (*w*/*w*). The CCRD consisted of 11 assays, as a function of temperature (T: 44–66 °C) and FA/AO molar ratio (MR: 1.2:1–6.8:1). Results were computed with Statistica software, version 10 (TIBCO, Tulsa, CA, USA). The influence of the factor’s temperature and substrate molar ratio on the response (C10:0 incorporation) was calculated, considering the linear and quadratic effects as well as their linear interaction. Analysis of variance evaluated their significance, being considered statistically significant at level 0.05 (*p* < 0.05). A three-dimensional surface, characterized by a second-order polynomial equation, was adjusted to the C10:0 incorporation values obtained in CCRD experiments [35]. The statistical principle of least squares was used to estimate the first and second-order coefficients of this equation from the experimental data. The determination coefficients (R^2^) and adjusted R^2^ (Radj^2^) were used to evaluate the goodness of fit of the model. To validate the model, the acidolysis of AO with C10:0, catalysed by RM IM lipase, was carried out for 24 h under the optimized conditions dictated by the model.

### 2.5. Batch Operational Stability Assays

Operational stability tests comprised eight successive 24 h batch reactions, at the predicted optimal conditions: 66 °C and a molar ratio of 4:1 (FA/AO). At the end of each batch, the lipase was removed by paper filtration, at room temperature, hydrated with 50 mL of a solution of phosphate buffer (pH 7; 0.1 M) and dried by filtration under vacuum to remove the excess buffer solution. Then, the recovered lipase was added to a fresh medium and reused in a new batch under the same reaction conditions. The catalytic activity of the lipase was considered as the observed molar incorporation degree of capric acid in argan oil TAG, at the end of each batch. The activity of the biocatalyst exhibited at the end of the first batch is considered 100% activity. After each reuse, the residual activity was expressed as a percentage of the activity in batch one.

The fit of the inactivation models was performed using the “Solver” add-in from Excel for Windows, by minimizing the residual sum-of-squares between the experimental results and those estimated by the models.

### 2.6. Reaction Products Analysis

All reaction products (FFA, partial acylglycerols and TAG) were separated by silica gel thin-layer chromatography (TLC). The TAG band was recovered, methylated and analysed by gas chromatography (GC), as fatty acid methyl esters (FAME), according to the methodology described by [36]. A gas chromatograph Finnigan TRACE GC Ultra (Thermo Electron Corporation) with a capillary column (60 m × 0.25 mm ID × 0.25 µm film) Thermo TR-FAME, and an AS 3000 autosampler from Thermo Electron Corporation were used for FAME analysis. The flame ionization detector (FID) was set at 280 °C and supplied with air and hydrogen at 350 and 35 mL min^−1^, respectively, The injector (in splitless mode) was set at 250 °C. The temperature program of the chromatographic column was the following: 100 °C for 1 min, increase to 160 °C at 10 °C min^−1^, held for 10 min, increase to 235 °C at 4 °C min^−1^ and kept for 10 min. The carrier gas Helium was supplied at a flow rate of 1.2 mL min^−1^. Fatty acid methyl ester mixes (PUFA No1 and PUFA No 3, from Supelco) were used as external standard and methyl myristate (C14:0) as internal standard.

The incorporation degree (*ID*) of C8:0 or C10:0 in TAG of argan oil, was calculated as follows, Equation (1) [20]:

(1) $$ID\;\left(\%\right)=\left(\frac{\mathrm{MFA}}{\mathrm{MT}}\right)\times 100$$

where MFA represents the C8:0 or C10:0 moles in the TAG and MT is the total amount of fatty acids (moles) in the TAG.

## 3. Results and Discussion

### 3.1. Fatty Acid Composition of Argan Oil

The fatty acid composition of argan oil used in this study is presented in Table 1, as well as the reference values following the Moroccan norm (N.M. 08.5.090), established for edible virgin argan oil.

The fatty acid profile of argan oil used in the present study fulfils the requirements established by the Moroccan norm. Oleic acid is the major fatty acid (41.1 ± 2.90), followed by linoleic (35.9 ± 1.40), palmitic (14.31 ± 0.63) and stearic (6.18 ± 0.19) acids.

### 3.2. Biocatalyst Screening

The commercial immobilized lipases Novozym^®^ 435, Lipozyme^®^ TL IM and Lipozyme^®^ RM IM were tested as catalysts for the acidolysis reaction between AO and C8:0 (system I) or C10:0 (system II) fatty acids, to produce MLM-type structured lipids. The tested enzymes were selected due to their (i) *sn*-1,3 regioselectivity, (ii) high activity in both aqueous and non-conventional media (aqueous/organic), (iii) ability to operate either in batch as in continuous reactors, (iv) stability at a wide range of pH and temperature, (v) operational stability, dependent on the reaction conditions and (vi) application at large-scale production. [4,9]. However, some studies indicate that Novozym^®^ 435 may present non-specific regioselectivity, depending on the reaction medium composition or immobilization supports [36,37,38,39].

The structured lipids (MLM type) were synthesized in a solvent-free medium which has several advantages, namely: enables its direct incorporation in food matrices without the need for solvent removal and SL purification, reduces the production costs, besides being a more environmentally friendly process. Additionally, the presence of an organic solvent will dilute the reaction medium leading to lower yields in MLM, even when high incorporation levels of medium-chain fatty acids are observed.

**Figure 1:**
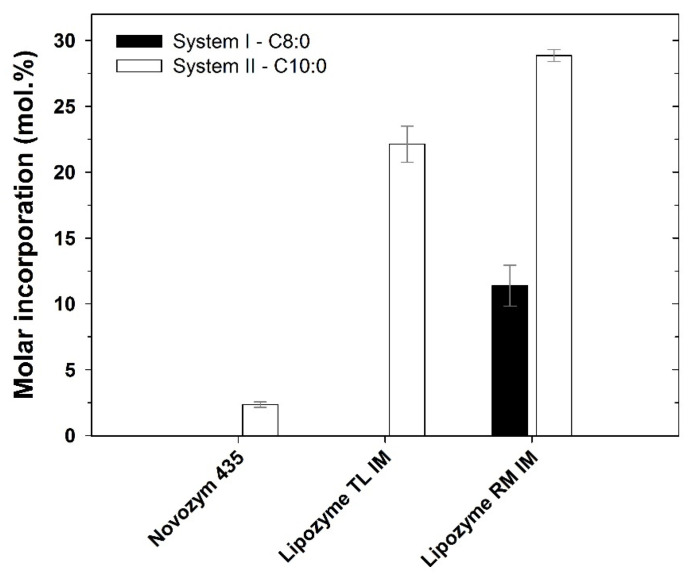
Molar incorporation of caprylic acid (System I. C8:0) or capric acid (System II C10:0) in the argan oil, using different commercial biocatalysts, at 55 °C after 24 h acidolysis in a solvent-free medium.

Results from the initial screening, to evaluate the most suitable biocatalyst, are presented in Figure 1. In this study, Lipozyme^®^ RM IM achieved the highest incorporation of C10:0 (28.9 ± 0.05 mol.%), being the only biocatalyst able to integrate C8:0 in argan oil, under the screening conditions (11.4 ± 2.2 mol.%).

Nunes et al. [23] used a similar approach for evaluating both C8:0 or C10:0 incorporations into virgin olive oil, catalysed by the same commercial immobilized lipases used in the present study. Two different reaction systems were tested (solvent vs. solvent-free medium) and higher incorporation degrees of C10:0 (ranging from 27.1 to 30.4 mol.%) were also observed in a solvent-free medium in comparison with C8:0 (19.9 to 25.7 mol.%), for all the biocatalysts tested. Low incorporations of caprylic acid into soybean oil (SBO), using Lipozyme TL IM as biocatalyst in the absence of organic solvent, were also reported by Turan et al. [40]. The highest yield (7.84%) was obtained at 40 °C, for an enzyme load of 10 wt.%, reaction time of 6 h and a molar ratio of 1:0.7 (SBO:C8:0). Kim et al. [41] reported higher incorporation of caprylic acid in perilla oil, by acidolysis catalysed by Lipozyme TL IM (30.6 mol.%) and by Lipozyme RM IM (34.2 mol.%), at 55 °C, using a molar ratio of 1:6, in solvent-free media. In the acidolysis reaction between C8:0 and avocado oil (rich in C18:1), catalysed by Lipozyme RM IM and Lipozyme TL IM, the highest incorporation value (29.2 mol.%) was attained for Lipozyme TL IM, operating at 30 °C along 24 h [12].

The type of oil, the biocatalyst and the operating conditions used are shown to greatly affect the success of acidolysis to synthesize low-calorie TAG. Considering the highest molar incorporation achieved with argan oil, the reaction system containing capric acid as acyl donor and using Lipozyme^®^ RM IM as catalyst was selected for subsequent studies.

### 3.3. Time-Course Reaction

**Figure 2:**
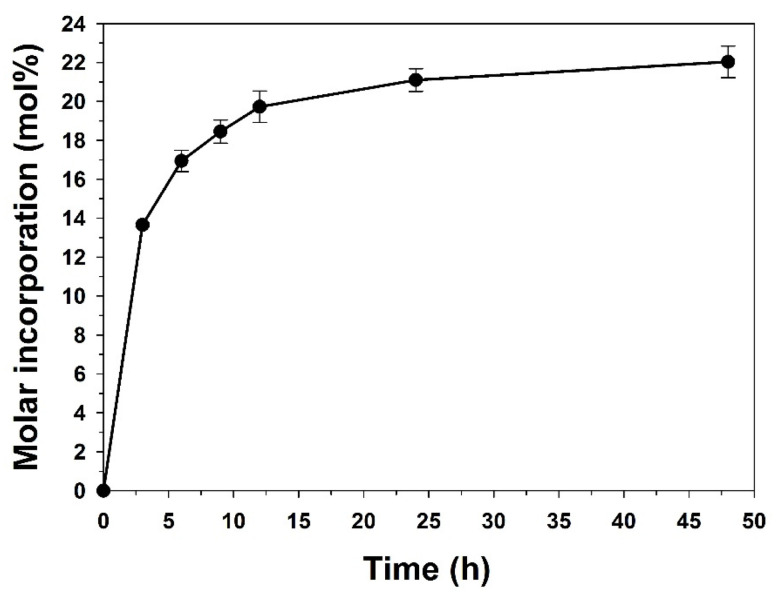
Incorporation (performed in duplicate) of C10:0 in the argan oil (molar ratio C10:0/AO of 2:1), catalyzed by 5% of Lipozyme^®^ RM IM (*w*:*w* of AO), in the absence of organic solvent, at 55 °C, during 48 h.

The evaluation of the enzymatic reaction kinetics is very important to select the time required to attain a quasi-equilibrium situation. Therefore, the incorporation of C10:0 in TAG from AO was monitored along time, under the same conditions followed for biocatalyst and system screening. The incorporation degree along 48 h acidolysis reaction is presented in Figure 2.

A quasi-equilibrium was observed between 24 and 48 h of reaction. After 24 h, C10:0 molar incorporation was averaged in 21.2 mol.% and 22.0 mol.% after 48 h acidolysis. Similar results of capric acid incorporation by acidolysis in solvent-free systems, were previously reported, with virgin olive oil (27.1 ± 2.3 mol.%) and Lipozyme RM IM after 24 h [23], with *Cucurbita maxima* pumpkin seed oil (29.9 ± 0.7 mol.%) and Lipozyme TL IM, at 45 °C, after 31 h acidolysis [42].

### 3.4. Modelling Acidolysis and Optimization of Reaction Conditions

In order to optimize the operational conditions that maximize capric acid incorporation in TAG of argan oil, the influence of the factors substrate molar ratio and temperature were evaluated through a set of acidolysis reactions following a CCRD. The experimental conditions and the obtained results are described in Table 2.

The highest incorporation degree (40.9 mol.%) was obtained at the highest temperature tested (66 °C) and a molar ratio to 4:1 C10:0/AO. Conversely, the lowest incorporation was observed for the lowest MR tested (1.2:1) which is below the stoichiometric value of 2:1. A negative or a positive linear effect of a factor (MR or T) on capric acid incorporation means a reduction or increase in the response, with the increase in the factor value, respectively. A positive or negative quadratic effect indicates that the response is described by a concave or convex response surface, respectively. The influence of MR and T on the acidolysis reaction was studied by evaluating both linear and quadratic effects of each factor, as well as their linear interaction on C10:0 incorporation in AO (Table 3).

The incorporation of C10:0 was influenced by both molar ratio and temperature. A positive linear effect was observed for temperature, being statistically significant (*p* = 0.043). This suggests that increasing temperature will result in increased incorporations. In fact, a temperature increase promotes the conversion of substrates into products by improving substrates solubility, decreasing reaction medium viscosity and favouring mass transfer [43,44]. The linear effect observed for MR was also positive and significant, suggesting the same outcome as for temperature. The quadratic effect of MR is negative with a *p*-value of 0.135, which is above the limit of 0.05. However, this effect must be considered because its removal would lead to a lack of fit of the polynomial model adjusted to the experimental data, which would be confirmed by a considerable decrease in both R^2^ and R^2^adj of that model. In fact, is preferred to accept factors presenting effects with *p*-values higher than 0.05 rather than to ignore some important factors [45]. The negative quadratic effect of MR indicates a convex response surface describing the incorporation of C10:0 in AO, as a function of MR. The interaction effect of T X MR is not significant (*p* = 0.503) and, therefore, can be ignored in the response surface model. In this study, C10:0 incorporation in the TAG followed a convex surface as a function of the molar ratio (indicated by the negative MR quadratic effect).

**Figure 3:**
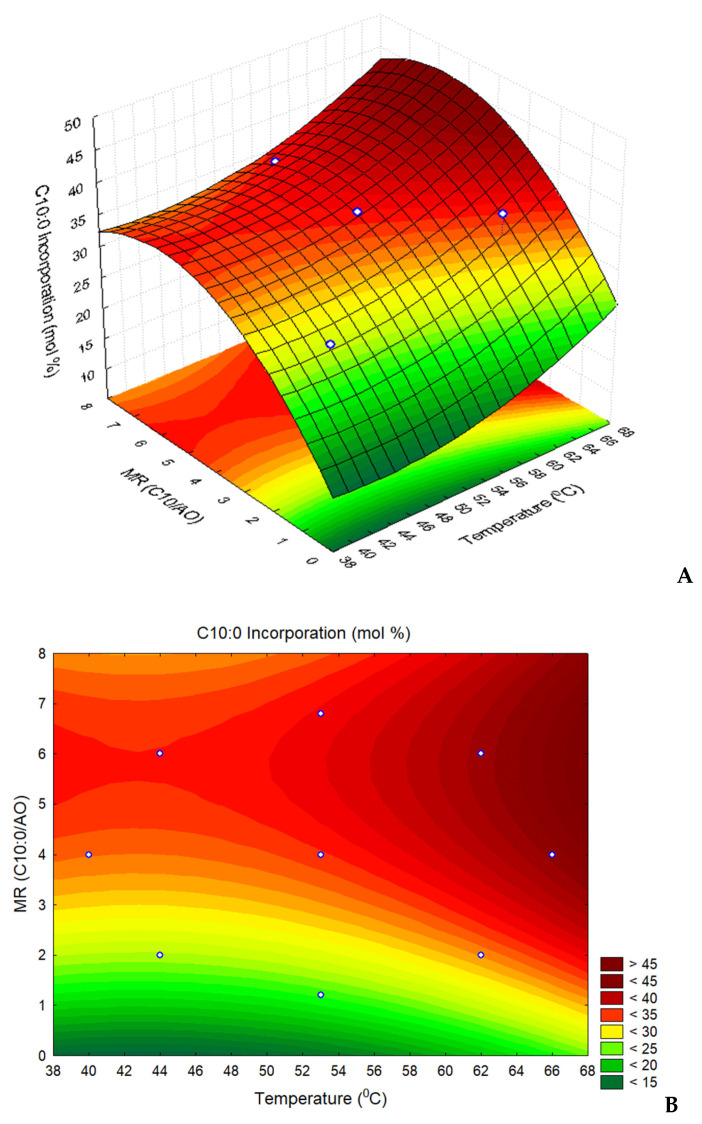
Three-dimensional response surface (**A**) describing the incorporation of capric acid (mol.%) in the argan oil, and (**B**) respective projection, as a function of reaction temperature and the molar ratio, MR, capric acid/argan oil (C10:0/AO).

The incorporation of C10:0 in TAG of AO, *ID*, as a function of both T and MR, catalyzed by Lipozyme^®^ RM IM, may be represented by a convex surface (Figure 3), described by the following second-order polynomial Equation (2):

(2) $$D\;\left(\mathrm{mol}.\%\right)=44.92-1.48T+0.017\;T^{2}+7.52\;\mathrm{MR}-0.649{\mathrm{MR}}^{2}$$

The values of the coefficient of determination (R^2^) and the adjusted coefficient of determination (R^2^_adj_) were 0.835 and 0.725, respectively. This shows a good fit of the model to the experimental data, proving them to be predictable by this model.

Figure 3 shows that the incorporation of C10:0 in argan oil increases with T but presents an optimal MR outside the experimental domain chosen in this study. Yet, it is possible to identify the region corresponding to the best results (T = 64–66 °C; MR = 4–5:1). In the acidolysis reaction between C8:0 or C10:0 and grapeseed oil, in the solvent-free system, using the same immobilized commercial lipases, Bassan et al. [19] reported an optimal temperature of 69 °C in the reaction system, also found by Response Surface Methodology, which was very close to the optimal temperature observed in this study (Figure 3). Additionally, the best results were reported for C10:0 as medium-chain fatty acid and Lipozyme RM IM lipase as a catalyst. High temperatures may compromise the activity and the operational stability of the enzyme (discussed in Section 3.5). The use of high MR in the reaction medium often presents no significant, or even a negative effect on SL synthesis, which may be attributed to an inhibition of the enzyme by the substrate and/or to a lipase deactivation due to the high levels of FFA in the reaction medium. This is sustained by several reports in the literature where increasing concentrations of FFA resulted in lower incorporations in the TAG backbone. Concerning the synthesis of human milk fat substitutes, lower incorporations of *n*3-PUFAs in the TAG structure of lard [26] and tripalmitin [46] were observed by increasing their concentration in the reaction medium, using the immobilized lipases of heterologous *Rhizopus oryzae* and *Carica papaya* as biocatalyst, respectively. Aiming at the production of low caloric TAG, [40] observed higher incorporations of caprylic acid into soybean oil at lower molar ratios, catalyzed by Lipozyme TL IM, being the highest value achieved at an MR of 1:0.7 (SBO:CA). Additionally, Nunes et al. [16] reported similar observations for the acidolysis of virgin olive oil with C8:0 or C10:0 fatty acids, catalysed by *Rhizopus oryzae*, with optimal molar ratios of 2.8:1 for C8:0/TAG and 3:1 for C10:0/TAG, respectively. These observations are aligned with the present results, reporting that increasing substrate molar ratio excessively may translate into lower yields. Such results are important to consider from an industrial point of view, since the use of large amounts of substrates will increase operational costs related to product and unconverted substrate recovery.

### 3.5. Operational Stability

**Figure 4:**
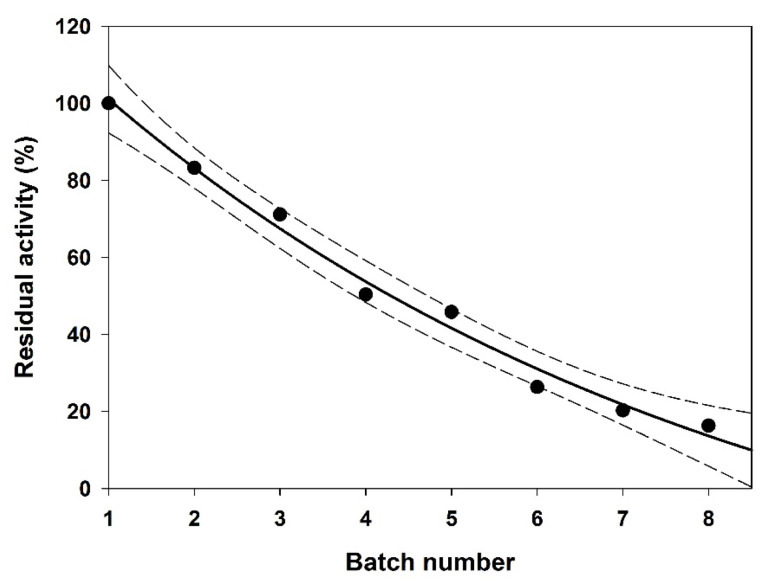
Lipozyme^®^ RM IM residual activity (%) at the end of each 24 h reuse, during acidolysis reaction between C10:0 and argan oil, at 66 °C and molar ratio of 4:1 (FA:TAG). Confidence intervals (95%) presented in dash lines.

The low operational stability is identified as one of the main drawbacks to the scale-up of enzyme-catalyzed processes to industrial applications. In this study, the operational stability assays were carried out at the best conditions predicted by the CCRD model, i.e., at 66 °C and a molar ratio of 4:1 (C10:0/AO). Eight consecutive 24 h batches were performed with Lipozyme^®^ RM IM, rehydrated between every two consecutive batches, accounting for a total of 192 operating hours. The results for residual activity after consecutive batches are presented in Figure 4.

Hydration favours the catalytic activity and prevents enzyme denaturation during dehydration in a non-aqueous reaction system [47]. Rehydration was carried out since it previously showed positive effects on the operational stability of r-ROL immobilized in Lewatit VP OC 1600, used as catalyst in the acidolysis of virgin olive oil with capric acid [24]. Conversely, negative effects of rehydration between batches on lipase activity, which was related to the enzyme itself and the carrier used for immobilization, have been reported [48].

A decrease in the biocatalyst residual activity was observed between each consecutive batch. After 96 h operation, Lipozyme RM IM retained 53% of its original activity. Deactivation results were best fitted to a first-order deactivation kinetics model, given by Equation (3).

(3) $$A_{n}=Ae^{-k_{d}n}$$

where *A_n_* is the residual activity (%) of the biocatalyst at the end of batch *n*, *A* is a constant that represents the enzyme initial activity before deactivation, and *k_d_* is the deactivation rate constant, expressed in *n*^−1^. The following Equation (4) represents the fit of this model to the experimental data:

(4) $$A_{n}=165.74e^{-0.131n}$$

Data showed a good fitting to the model (R^2^adj = 0.989), with a half-life time (t_1/2_) of 103 h, corresponding to approximately 4.3 days. These results can be compared to some reports in the literature, where similar operating systems and the same biocatalysts were tested. Lipozyme RM IM presented higher operational stability in (i) the continuous interesterification between palm stearin (45% *w*/*w*), palm kernel oil (30% *w*/*w*) and olive oil (25% *w*/*w*), using a packed-bed bioreactor at 65 °C [49], (ii) the interesterification of tripalmitin with oleic acid or omega-3 polyunsaturated fatty acids (omega-3 PUFA) as acyl donors, aiming at the production of human milk fat substitutes [36], and (iii) the synthesis of MLM structured lipids by acidolysis of virgin olive oil with caprylic or capric fatty acids [23].

Lipozyme^®^ RM IM is immobilized in a macroporous anion exchange resin, which confers thermal stability to the biocatalyst. However, extended operating periods at high temperatures may cause lipase inactivation and its release from the support, or the opposite situation, release from the support and consequent inactivation [50].

Although it is a thermostable lipase, when contrasting the present results with the literature, a credible better performance of Lipozyme RM IM at milder temperatures for more prolonged activity periods may be expected. This was previously reported for similar reaction systems [12,23]. This may also be related to the inactivation of the biocatalyst by lipid oxidation compounds formed at high temperatures, more than biocatalyst preferences for specific length FA. A cost-effective analysis of operating conditions (stability vs. temperature) would be necessary to predict the optimal reaction conditions for MLM production using Lipozyme RM IM as catalyst. It has also been reported that the purity of the substrates influences the stability of biocatalysts. Minor natural compounds in the reaction medium, such as phospholipids, emulsifiers, chlorophyll, carotenoids, or antioxidants, remarkably affect the stability of lipases [51]. Virgin argan oil contains about 1% of these molecules. Natural molecules such as tocopherols and polyphenols were not evaluated in the present study, but initially, their antioxidant activity would be expected in the reaction medium due to their biochemical nature, interfering with lipid autoxidation by a prompt donation of hydrogen atoms to lipid radicals [4]. However, the relatively high optimal temperature (66 °C), along with the reported negative effects for these compounds may have adversely affected the biocatalyst stability in this study. An additional characterization of these molecules in the oil, along with its oxidative stability, would be a compelling prospective goal.

## 4. Conclusions

This study showed that argan oil may be a feasible substrate for MLM-type structured lipid production. In fact, using this oil as TAG source in the acidolysis reaction with capric acid (C10:0), it was possible to achieve an incorporation degree of 40.9 mol.% at the optimal conditions dictated by the response surface methodology (66 °C and a molar ratio 4:1 C10:TAG), after 24 h. This was achieved by using Lipozyme RM IM, which showed to be the best biocatalyst comparatively to other commercial lipases tested in this reaction system. The biocatalyst presented a half-life time (t_1/2_) of 103 h, after eight consecutive 24 h batches, at the same reaction conditions. Operating conditions such as MR and temperature are important factors to consider in similar operating systems to further implementation at a larger scale.

## Figures and Tables

**Table 1:** Comparative analysis of fatty acid profile of argan oil used in the present study and the official values established in the Moroccan norm (N.M. 08.5.090). Samplings were performed in duplicate and results are presented in percentage, with standard deviation (S.D.). C represents the number of carbon in the fatty acid and D is the number of double bonds in the carbon chain; SFA: saturated fatty acids; MUFA: monounsaturated fatty acids; PUFA: polyunsaturated fatty acids.

Fatty Acid	Lipid Number (C:D)	Percentage (% ± S.D.)	Normative Composition (Charrouf and Guillaume 2014)
Myristic	C 14:0	0.20 ± 0.01	≤0.2
Palmitic	C 16:0	14.31 ± 0.63	11.5–15.0
Palmitoleic	C 16:1 n9	0.14 ± 0.01	≤0.2
Stearic	C 18:0	6.18 ± 0.19	4.3–7.2
Oleic	C 18:1, n9	41.1 ± 2.90	43.0–49.1
Linoleic	C 18:2 n6	35.9 ± 1.40	29.3–36.0
α-Linolenic	C 18:3 n3	0.125 ± 0.01	≤0.3
Arachidic	C 20:0	0.31 ± 002	≤0.5
Gadoleic	C 20:1 n9	0.34 ± 0.01	≤0.5
Behenic	C 22:0	0.1 ± 0.001	≤0.2
∑ SFA	-	21.2 ± 0.87	-
∑ MUFA	-	42.4 ± 2.34	-
∑ PUFA	-	36.27 ± 1.44	-

**Table 2:** Experimental matrix, as a function of molar ratio (MR, C10:0/AO) and temperature (T; °C), and the results obtained for molar incorporation of C10:0 in TAG from argan oil, after 24 h acidolysis catalyzed by Lipozyme^®^ RM IM.

Experiments	Coded Matrix		Decoded Matrix		Incorporation (mol.%)
	Temperature (°C)	Molar Ratio (C10:0/AO)	Temperature (°C)	Molar Ratio (C10:0/AO)	
1	−1	−1	44	2:1	27.7
2	−1	1	44	6:1	35.4
3	1	−1	62	2:1	36.9
4	1	1	62	6:1	39.7
5	−1.414	0	40	4:1	32.1
6	1.414	0	66	4:1	40.9
7	0	−1.414	53	1.2:1	19.0
8	0	1.414	53	6.8:1	37.8
9	0	0	53	4:1	34.7
10	0	0	53	4:1	37.2
11	0	0	53	4:1	32.5

**Table 3:** Linear and quadratic effects of molar ratio (MR), temperature (T)and linear interaction (T X MR) in the incorporation of C10:0 in argan oil. with the corresponding *p* values.

Factor	Effect	*p*-Value
T (linear)	6.41	0.043
T (quadratic)	2.81	0.357
MR (linear)	9.30	0.012
MR (quadratic)	−5.19	0.135
T X MR (linear)	−2.45	0.503
